# Neuronal HLH-30/TFEB modulates thermoresistance and longevity in *C. elegans*

**DOI:** 10.18632/aging.204849

**Published:** 2023-10-12

**Authors:** Louis R. Lapierre

**Affiliations:** 1 Département de Chimie et Biochimie, Université de Moncton, Moncton, NB E1A 3E9, Canada; 2 Department of Molecular Biology, Cell Biology and Biochemistry, Brown University, Providence, RI 02912, USA; 3 New Brunswick Center for Precision Medicine, Moncton, NB E1C 8X3, Canada

**Keywords:** HLH-30/TFEB, aging, heat stress response, *C. elegans*, neuronal signaling

## Abstract

The conserved autophagy transcription factor HLH-30/TFEB is a well-established modulator of lifespan in several mechanistically-distinct longevity paradigms in *C. elegans*. While various tissues contribute differentially to organismal lifespan, neurons are particularly interesting as they can mediate adaptive response to environmental and proteostatic stresses. Using carefully-designed neuronal-specific reconstitution of HLH-30 in loss of function *hlh-30* mutants, we found a role for neuronal HLH-30 in modulating longevity and heat stress response via neurotransmission-mediated peripheral mitochondrial fragmentation. Altogether, we demonstrated new links between neuronal HLH-30 function, thermoresistance and organismal aging.

The conserved autophagy transcription factor HLH-30/TFEB is a well-established modulator of lifespan in several mechanistically-distinct longevity paradigms in *C. elegans*. While various tissues contribute differentially to organismal lifespan, neurons are particularly interesting as they can mediate adaptive response to environmental and proteostatic stresses. Using carefully-designed neuronal-specific reconstitution of HLH-30 in loss of function *hlh-30* mutants, we found a role for neuronal HLH-30 in modulating longevity and heat stress response via neurotransmission-mediated peripheral mitochondrial fragmentation. Altogether, we demonstrated new links between neuronal HLH-30 function, thermoresistance and organismal aging.

HLH-30 is an orthologue of the transcription factor EB (TFEB) of the microphthalmia transcription factor family (MITF) and modulates the expression of conserved autophagy genes from *C. elegans* to humans [1]. Loss of *hlh-30* leads to reduced proteostasis and autophagy [1], decreased thermoresistance and ability to counteract pathogenic infections [2], and an impaired capacity to sustain lifespan extension associated with adult reproductive diapause [3] and longevity [1]. In *C. elegans,* the localization and activity of HLH-30 is modulated by the major nutrient sensing mTORC1 complex [1, 4] and the nuclear export protein XPO1 [5, 6].

Nuclear localization of longevity-associated transcription factors is a feature of many long-lived models in *C. elegans*, including the quintessential insulin/IGF-1 receptor *daf-2* mutants [7]. In the nucleus, HLH-30 can interact with various proteins and transcription factors to generate different transcriptional signatures. For instance, the interactions between HLH-30/TFEB and DAF-16/FOXO lead to context-dependent improvements in survival and stress resistance [8]. Despite these important discoveries of HLH-30’s systemic functions, the tissue-specific roles of HLH-30 was not yet established. In Wong SQ et al. [9], we sought to determine this in the context of thermoresistance and longevity, specifically in neurons, where organism-wide regulation of stress responses can originate.

Unexpectedly, reconstitution of neuronally-expressing HLH-30 in *hlh-30* mutants was not reproducibly successful at restoring normal lifespan, possibly due to its general cytoplasmic localization [1]. However, re-introducing HLH-30 in the neurons of *daf-2;hlh-30* mutants showed significant improvements in lifespan, suggesting that neuronal HLH-30 is important for longevity where animals have HLH-30 enriched in the nucleus [1]. Surprisingly, the importance of functional HLH-30 in neurons of *daf-2* for lifespan extension did not translate to its requirement in thermoresistance. Indeed, loss of *hlh-30* in *daf-2* animals did not substantially affect their ability to withstand heat stress, suggesting compensatory mechanisms. The opposite was true in *hlh-30* mutants where neuronal reconstitution of HLH-30 had a significant impact on their thermoresistance. This provided clues that thermoresistance and longevity are not completely overlapping mechanistically.

Transcriptomic analyses of animals under heat stress in the absence or presence of neuronal HLH-30 revealed differential regulation of several genes, one of which, W06A11.1, was required for thermoresistance in neuronal HLH-30-rescued animals. As heat stress elicit peripheral mitochondrial fragmentation, we found that W06A11.1 is necessary to modulate this mitochondrial dynamic change. Notably, connections between neuronal signaling modulated by the mTORC1 complex and mitochondria morphological dynamics were previously demonstrated [10]. Although annotated as peripherally expressed on Wormbase, we observed that fluorescently-tagged W06A11.1, when expressed under its endogenous promoter, is induced specifically in the head of *C. elegans* in the presence of heat stress. Interestingly, we found that HLH-30 and W06A11.1 modulate peripheral mitochondrial fragmentation through neurotransmission, uncovering a new player in neurons that can enact systemic changes in stress response (Figure 1). Here, we propose that targeting neuronal HLH-30/TFEB may impact these signals to enhance stress resistance and lifespan.

**Figure 1:**
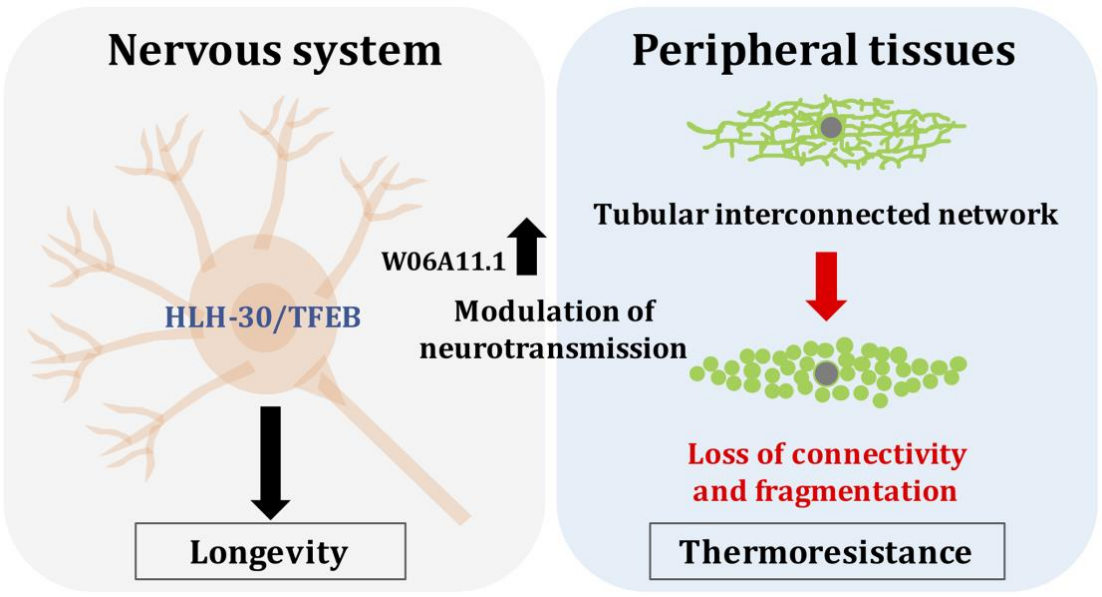
Neuronal HLH-30 is necessary for longevity in *daf-2* animals and W06A11.1 modulate peripheral mitochondrial fragmentation to enhance thermoresistance in wild-type animals.
